# Effect of Modification of the NI Artificial Diet on the Biological Fitness Parameters of Mass Reared Western Tarnished Plant Bug, *Lygus hesperus*

**DOI:** 10.1673/031.011.14901

**Published:** 2011-11-08

**Authors:** Maribel Portilla, Gordon Snodgrass, Doug Streett

**Affiliations:** ^1^ United States Department of Agriculture, Agricultural Research Service, USA; ^2^ United States Forest Service, Southern Research Station, USA

**Keywords:** fitness estimate, *Lygus* diet, mass rearing, quality control

## Abstract

The NI artificial diet is the only known successful diet for mass rearing the western tarnished plant bug, *Lygus hesperus* Knight (Hemiptera: Miridae). This diet has been used for more than a decade. However, because it contains cooked chicken egg, and thus requires laborious preparation (Cohen 2000), this diet is difficult to use. Three modifications (D1, D2, D3) of the NI diet were investigated in hopes of developing a more easily prepared diet that avoids the cooked egg and improves mass fitness parameters of *L. hesperus*. The modified D3 diet, containing autoclaved chicken egg yolk based component, had the highest egg/cage/day production (13120 ± 812 SE). This was significantly greater than diets D1, containing autoclaved dry chicken egg yolk based component (9027 ± 811 SE), D2, containing autoclaved chicken egg white based component (8311 ± 628 SE), and NI, which contained autoclaved chicken egg yolk + cooked egg diet (7890 ± 761 SE). Significant differences were observed in the weights of all developmental stages except for eggs and first instar nymphs. Higher rates of fertility, hatchability, and low mortality in nymphs during the first instar were also obtained in the modified D3 diet. The results clearly indicated that the D3 diet provided an opportunity to significantly reduce rearing cost by avoiding time-consuming issues with preparation of a cooked egg diet. This should result in an increase in production capacity and a reduction in production costs.

## Introduction

Western tarnished plant bug, *Lygus hesperus* Knight (Hemiptera: Miridae), is a destructive pest that attacks many important crops including alfalfa (*Medicago sativa*), beans (*Phaseolus vulgaris*), strawberries (*Fragaria virginiana*), peaches (*Prunus persica*), cotton (*Gossypium hirsutum*), and various other seed crops. Its impact is amplified by a remarkable ability to become resistant to pesticides (Hedlund and Graham 1987). Development of management programs based on biological alternatives such as biological control, biorational chemicals, plant breeding, sterile insect release, and genetic engineering of target crops is very important and all these approaches depend on a mass rearing system (Cohen 2000). Rearing of *Lygus* on artificial media began when Auclair and Raulston (1966) reported limited success rearing *L. hesperus* on a chemically defined diet. Vanderzant (1967) and Frank and Krutwagen (1969) developed diets that supported growth and development of *L. hesperus* comparable to rearing them on fresh green beans. The Debolt diet (Debolt 1982) and more recently the NI diet (Cohen 2000) have been the only two diets that supported several generations. The NI diet, however, is the only diet that has been successfully used for mass rearing of *L. hesperus*.

The disposable diet packet used for feeding and oviposition of *L. hesperus* and *L. lineolaris* (Palisot de Beauvois) developed by Patana (1982), plus the NI artificial diet developed by Cohen (2000), have allowed for the production of millions of mirids for biological, chemical, and genetic studies. For years, several commercial insectaries and research agencies have used the NI diet for the continuous production of *L. lineolaris* and *L. hesperus*. However, because preparation of the diet is time consuming, its use is not particularly widespread. Use of green beans or other natural hosts are more convenient and are frequently used to rear *Lygus*.

This investigation aimed to improve upon the semisolid artificial NI diet by avoiding the cooked egg component, and eliminating other components such as antibiotics, acids, and formaldehyde without affecting the mass fitness parameters of the *L. hesperus* produced.

## Materials and Methods

### Tarnished plant bug colony

This study was conducted at the USDA Agricultural Research Station NBCL, located in Stoneville, MS. Adults were from a colony established in 1998 (Cohen 2000) and maintained previously in the USDA Agricultural Research Station BCPRRU, in Starkville, MS. The colony was moved to the NBCL facility in 2003. *L. hesperus* was reared according to the method described by Cohen (2000) with some modifications; both sides of the gel pack were used for oviposition rather than only one side, parafilm with the attached eggs was removed from the gel pack and placed on the surface of the shredded paper, and non-stretched feeding packs were used inside the rearing container. The standard cages used for all life stages were Rubbermaid 8.3 1 rectangular storage boxes (Rubbermaid, www.rubbermaid.com), with 15 × 25 cm openings cut into the bases and covered with bleached muslin fabric (www.theonlinefabricstore.com). The top was also cut and the 20 × 30 cm opening was replaced with bleached muslin fabric for smaller nymphs, and changed with 1.0 mm mesh fiberglass screen for larger nymphs and adults. The insects were reared in an environmental room with a photoperiod of 16:8 L:D, temperature of 27° C (± 1.5° C), and 55% relative humidity, ± 10% (Cohen 2000).

#### Diet Preparation

The NI diet consisted of three groups (A, B, C), while the diet modifications (D1, D2, D3) consisted of two groups (B, C) (Table 1). The NI diet was prepared according to the procedure of Cohen (2000). The cooked egg diet (Group A) was made by mixing water, sucrose, honey, and acetic acid (see Table 1 for amounts), then bringing the mixture to a rapid boil, at which time whole chicken eggs were added, stirred, and heated until the mixture had scrambled egg consistency. The Group B components—toasted wheat germ, coarsely ground lima bean meal, soy flour, tap water, and egg yolk—were mixed and autoclaved for 20 minutes at 120° C and 15 1b/m^2^. Group C consisted of water, formaldehyde, soy lecithin with oil, Vanderzant vitamin mixture, propionic acid, Chlortetracycline, and streptomycin sulfate; ingredients were weighted, mixed, and blended for four minutes with groups A and B once they had cooled to ∼ 50° C. Groups B and C of the diet modifications D1, D2, and D3 were prepared following the procedures of NI (Groups B and C) diet preparation.

#### Mass fitness parameters

**Effect of diet modification on the reproduction rates**. To compare the mass fitness parameters for each diet, nymphs in a rearing cage were fed one of the treatment diets (D1, D2, or D3) while nymphs in the control were fed the standard NI diet. Approximately 3000 first-instar *L. hesperus* were placed in each cage in each of the three replications of the four treatments. The first day of oviposition by an emerged adult occurred after approximately 18 days of rearing. One diet parafilm pack per cage was used for *L. hesperus* feeding and one gel parafilm pack per cage was used for oviposition (Cohen 2000). Both diet and gel packs were removed every 24 hours. The gel egg packs obtained from each cage/treatment were placed in a tray and kept for five days. On the sixth day the edges of the gel pack were cut and the parafilm with the attached eggs was removed from the gel and placed into a sealed plastic rearing container measuring 3 cm depth × 15 cm diameter. This container was ventilated and was originally developed for use in the mass production of *Phymastichus coffea* (Portilla and Streett 2008). Eggs were held for 24 hours in the container for egg hatch. After the 24-hour period, data was collected, including total eggs, number of undeveloped eggs (no embryo), infertile eggs (white embryo), eclosion percentage (hatchability), and total mortality of first-instar *L. hesperus* at the time of emergence. These data were collected daily for eggs laid in each replication of each treatment for 10 days after oviposition began. At the end of the 10 day period, live females were counted and the number was divided into the total number of eggs oviposited each day to calculate eggs/day/female. The Image ProPlus software for Windows (www.mediacy.com) was used to count and calculate egg production.

#### Effect of diet modifications on stage weight.

To calculate the effect of the modified diets on the mean weight of various stages of *L. hesperus*, three replicate cages of the D1, D2, D3, and NI diet treatments were set up with F_1_ progeny (first instar) using the same procedure described above. As the F_1_ progeny developed, 30 individuals per stage (egg, first and fifth instar, adult female, and adult male) were randomly taken from each replication of each treatment and individually weighed. A Santorium ISO 9001 microbalance (www.sartorius.com) was used to determined weight in each stage.

**Figure 1.**
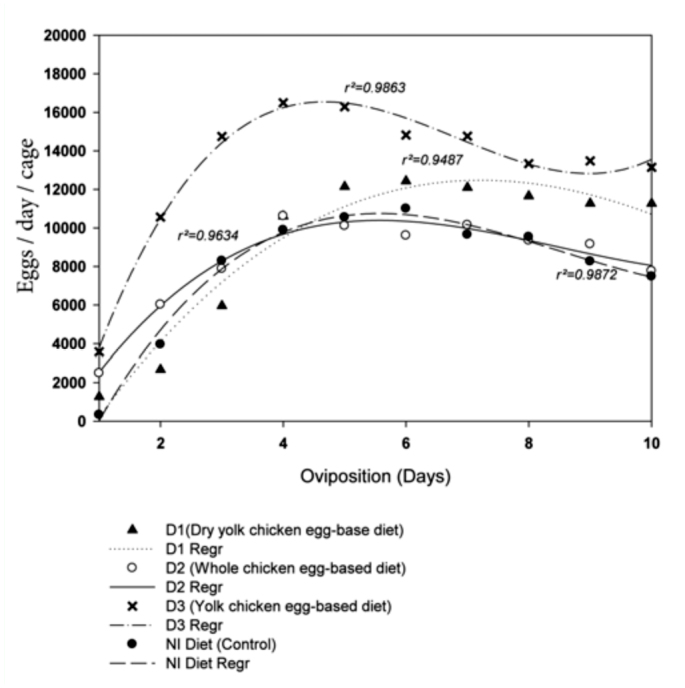
Daily egg production by *Lygus hesperus* on the NI diet and three modified NI diets (D1, D2, D3). High quality figures are available online.

##### Statistical analysis

Parametric statistics were used to compare means of egg production and weight of first and fifth instar, along with female and male adults. Nonparametric statistics for rates of fertility, hatchability, first instar mortality, and daily egg production per female were performed using SAS system software (SAS Institute 2008). Data for progeny per rearing cage, fertility and hatchability rate, first instar mortality rate, and maximum weight were analyzed using analysis of variance with the PROC GLM procedure of SAS. Differences in treatment means were detected using Tukey's test (*p* < 0.05). Regression analysis was used to determine correlations between diet modifications and egg production rate per day.

**Figure 2.**
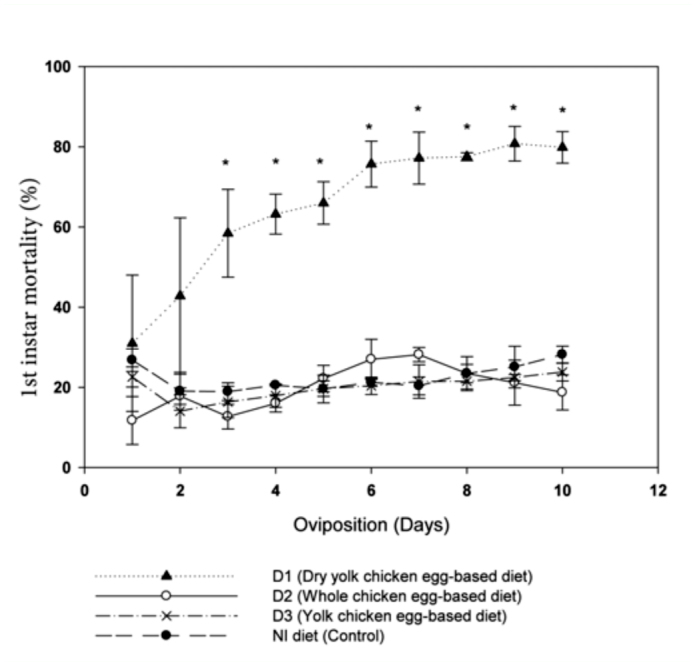
Mortality of the first instar *Lygus hesperus* reared on NI artificial diet and three NI diet modifications (D1, D2, D3). (*) indicates that mortality in D1 diet was significantly higher than mortality in the other three diets on that date (Tukey's HSD test, *p* = 0.05). High quality figures are available online.

#### Results

Figure 1 shows the regression analysis performed (cubic trend model) with egg production rate per day. The only trend significantly different from the trend for the NI control diet was the trend for the D1 diet (Table 2). Egg production on the D1 diet began at a lower rate but increased from day five to day seven before production began to decline. During this time period egg production in the other three diets was declining. Table 2 shows the equations for the trends and the actual egg rate based on the cubic trends for days three, four, five, and six.

**Figure 3.**
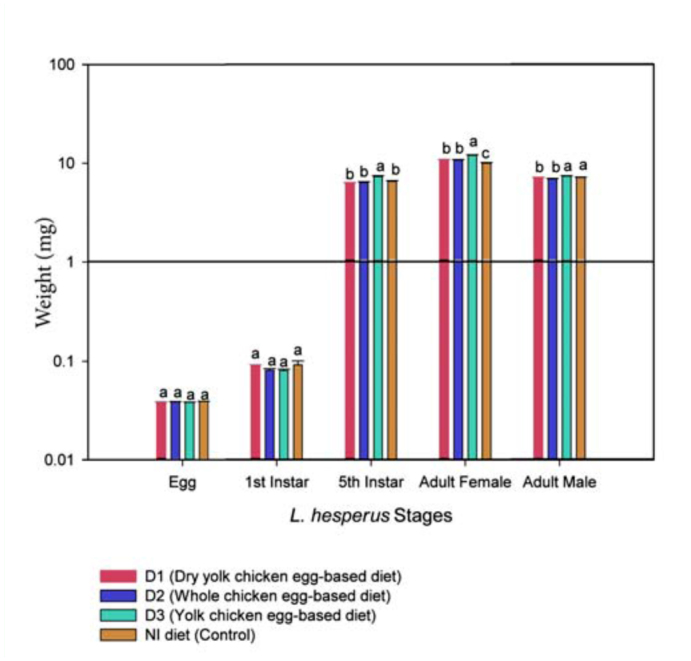
Effect of NI diet modifications on the maximum weight of various stages of the *Lygus hesperus*. For any given stage, mean (± SE) followed by the same letter were not significantly different (One way ANOVA and Tukey's HSD test *p* = 0.05). High quality figures are available online.

Mortality in the first instar (at the time of hatching) was much higher when *L. hesperus* parents were reared on D1, the dry-yolk chicken egg-based diet. Mortality of nymphs fed the D1 diet increased every day as compared to mortality to the NI, D2, and D3 diets, in which mortality was consistently lower (Figure 2). However, nymphs that survived on the D1 diet did not differ in size or weight in the fifth instar and as adults when compared with the insects reared on the NI or D2 diets (Figure 3). In general, *L. hesperus* developed normally on all diet modifications. Table 3 shows that there were no significant differences between the NI control diet and all three NI diet modifications in percentage of undeveloped eggs (no embryo)/day/gel pack, infertile eggs (with embryo)/day/gel pack, hatchability rate/day/gel pack, and total survival of females after 10 days of oviposition/cage. The mean daily egg production per gel pack was significantly higher on the modified diet D3 than in the D1, D2, and NI diets with means of 13,120 ± 802, 9027 ± 811, 8,311 ± 628, and 7890 ± 761 eggs/day/gel pack, respectively (*F* = 9.14; df = 3, 2; *p* < 0.01). Similar results were also obtained with two other important fitness measures; first instar mortality/day/gel pack was significantly higher (*F* = 62.00; df = 3, 2; *p* = < 0.01) using the D1 diet as compared to the others three diets (Table 3), and total eggs/day/female was significantly higher (*F* = 3.11; df = 3, 2; *p* = 0.0113) using the D3 diet compared with NI diet, but not with the D1 and D2 diets.

Maximum weights for eggs, first and fifth instars, and female and male adults of *L. hesperus* are provided in Figure 3. Results indicated significant differences between diets in weight in the fifth instar (*F* = 19.21; df = 3, 2; *p* = < 0.01), female (*F* = 34.81; df = 3, 2; *p* = < 0.01), and male (*F* = 6.54; df = 3, 2; *p* = < 0.01) stages, though no differences were found for eggs or first instar stages. Upon reaching the fifth instar *L. hesperus* females and males were always significantly larger in weight for individuals reared on the D3 diet. Daily egg production and greater live weight for female, male, and fifth instar stages obtained with the D3 diet resulted in greater biomass accumulation.

### Discussion

Slight differences between diet components can have an effect on feeding preferences (Singh and Moore 1985), which can affect the required criteria for evaluating nutritional adequacy of a diet used for mass rearing. Two of the most important indicators of a quality diet are biomass accumulation and fecundity (Cohen 2000). Our results indicated that the modified D2 and D3 diets were adequate for development and mass production of *L. hesperus*. However, the D3 modified treatment was superior in biomass accumulation and fecundity to the regular NI diet (control). Egg production with the D3 diet (eggs/gel pack/day) was similar to that found by Cohen (2000), which determined the fecundity of *L. hesperus* with the regular NI diet. Cohen (2000) also found that fecundity was very poor when using the Debolt diet compared with the NI diet. Fecundity with the NI diet in our three modifications (D1, D2, D3) was also higher than that obtained with the Debolt diet by Cohen (2000).

The greater biomass accumulation in the D3 treatment was associated with a significantly greater egg production of 13120 ± SE 812 eggs/gel pack/day (Table 3). Portilla et al. 2010 (In press) found that from the total egg production, only 34% of insects from the modified NI diet (D3) and 33% from the regular NI diet (control) became adults, with a sex ratio of 1.12♀: 1♂ and 1.18♀: 1♂, respectively. They also found that about half of that population (D3: 51.11%, NI diet: 48.33%) was still alive 10 days after the first day of oviposition. Therefore, the 24-hour D3 diet resulted in ∼ 4500 reared adults and ∼ 1100 live females 10 days after the first day of oviposition. In the NI diet (7890 ± SE 761 eggs/gel pack/day, Table 3), ∼ 2600 adults were reared and ∼ 600 females were alive 10 days after the first day of oviposition. The biomass accumulation obtained in the NI diet (control) was similar to Cohen 2000, who obtained roughly 1000 adults per standard cage (500 females) of *L. hesperus* from a 5000-egg gel pack. One large factor in the extremely low adult survival obtained in this study and in studies by Cohen (2000) and Portilla et al. (2010) was cannibalism in the nymphal and adult stages, especially at high rearing densities. *L. hesperus*, like other *Lygus* spp., is an omnivore that consumes both plantbased foods and arthropod prey (Rosenheim et at. 2004). Omnivory in *L. hesperus* has been documented in both the laboratory and field (Wheeler 1976 and 2001, Cohen 1996).

It is important to note that the live weight was greater for fifth instar, along with female and male adults of *L. hesperus* reared with the D3 diet than it was for those reared with the D1, D2, and NI diets. The higher live weight per individual female was related to body size and fecundity as shown in Figure 3 (female mg) and Table 2 (eggs/day/female): D1 (10.79 mg: 11.74 eggs), D2 (10.73 mg: 10.36 eggs), D3 (12.02 mg: 12.51 eggs), and NI (9.99 mg: 8.62 eggs). The higher live weight could also be attributed to the nutrient composition. Cohen (2000) mentioned that the regular NI diet contained 6% protein, 2% lipid, 17% carbohydrates, and 75% water. The NI diet modifications we tested had almost the same components, but differed in diet preparation by avoiding cooking part A (cooked egg diet). This could have minimized any detrimental changes to diet ingredients such as vitamins and minerals that are present mainly in the yeast. Yeast was exposed to a boiling point for several minutes in the regular NI diet preparation together with sucrose, honey, and acetic acid solution (Cohen 2003). Heating should be at the lowest practical temperature and the shortest time to avoid breakdown of some ingredients (Vanderzant 1969). In the diets we tested, the Brewer yeast was replaced with Torula yeast, *Candida utilities*. Portilla and Streett (2008) stated that 50% of this yeast was protein with the remaining 50% made up of ash (< 8%), salts (< 0.5%), vitamins (thiamine, riboflavin, niacin), lipids, minerals, and other substances that stimulate feeding responses mainly in immature stages. This may explain why the *L. hesperus* females reared on the three diet modifications had higher live weight than those reared on the regular NI diet. Similar results were found by Portilla et al. (2010), who obtained higher live weight in females and males of *L. lineolaris* fed with the D3 diet. Chang (2009) reported greater pupal weight for the oriental fruit fly, *Bactrocera dorsalis* (Coquillet) fed with torula yeast after comparison with insects fed with Brewer's yeast and Korea yeast.

The efficacy of using cooked eggs or raw eggs was discussed by Cohen and Smith (1998), who found it to be a necessary component for mass rearing in *L. hesperus* and *L. lineolaris.* It is not an indispensable ingredient for completion of the life cycle of *L. hesperus*, as we demonstrated in our study with the dry yolk-based D1 diet. The use of egg yolk autoclaved with plant material (component B in D3) seemed to be essential to the nutritional and phagostimulatory quality of the complete diet as mentioned by Cohen (2000). However, we demonstrated that using diet enriched with extra yolk was not necessary to satisfy the dietary need of *L. hesperus* and *L. lineolaris* for animal derived material, as cited by Cohen (2000). The use of fresh chicken egg should be considered a drawback for any *Lygus* diet.

Mold control in insect diets and colonies is one of the greatest challenges of insect rearing (Singh and Moore 1985). The temperature and humidity under which *L. hesperus* is reared are ideal for mold growth. *Aspergillus niger* is one of the most commonly encountered contaminants in *Lygus spp*. colonies, and is also one of the most difficult to control (Alverson and Cohen 2002). Antimicrobials are frequently added to artificial diets, often without taking into account possible detrimental effect on insect and human health. Portilla and Streett (2008) demonstrated that formalin (1 ml/L of diet) could be substituted for benzoic acid (1.5 g/L of diet) in artificial diets, such as Cenibroca and MP diets, for rearing *Hypothenemus hampei* and its parasitoid *P. coffea*. Alverson and Cohen (2002) evaluated the effect of antifungal agents on the biological fitness of *L. hesperus*. They found that benzoic acid (2000, 3000, 4000 ppm), propionic acid (800, 1200, 1600 ppm), and sorbic acid (800, 1200, 1600 ppm) did not significantly affect biological fitness as compared with the control at any of the three concentrations. However, the highest total biomass, measured as dry weight in mg, was obtained when insects were reared with the NI diet prepared with 2000 and 3000 ppm of benzoic acid. Based on these studies, we replaced the formaldehyde (1 ml), propionic acid (1 ml), and acetic acid (2 ml) in the three modified NI diets with benzoic acid (2 g). Bacteria contamination in *Lygus* diet is uncommon; the 25 mg of streptomicycin added to D1, D2, and D3 diets was used as a preventive compound.

We modified the regular NI diet in order to obtain an inexpensive diet that required little labor to produce. This was achieved by cutting the cooking part (referred to as NI cooked egg diet component A), reducing antibiotics, avoiding the addition of formaldehyde, and by changing other ingredients such as preservatives and minerals. These modifications made the NI artificial diet less expensive (20% reduction) (Table 1), less laborious (75% reduction), and more importantly, completely safe during preparation because of the removal of formaldehyde, a carcinogenic agent. The artificial modified D1, D2, and D3 diets were prepared in a single-step process, thereby avoiding the multi-step process used to prepare the NI diet. The modified NI artificial D3 diet permits large scale rearing of omnivore insects such us *L. hesperus*. This species can be produced with higher biological fitness values than those reported for the existing standard NI diet.

**Table 1.**
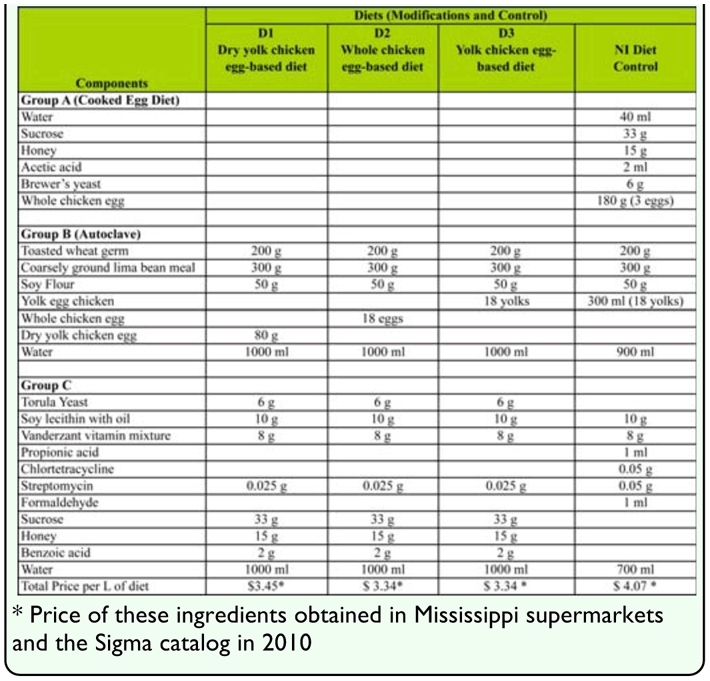
Diet components of NI artificial diet and three modifications (small batch: approximately 2.5 L of diet)

**Table 2.**
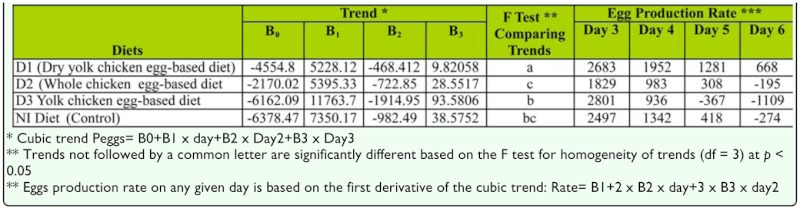
Cubic trend model egg production over time for *Lygus hesperus* reared on the NI Diet and and three modifications of the NI diet.

**Table 3.**
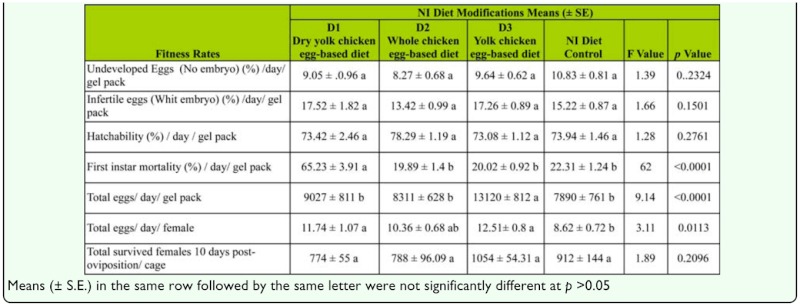
Effect of the NI diet and three NI diet modifications on fitness of *Lygus. hesperus*.
